# Peri-Implant Tissue Health and Prosthodontic Outcomes of a Cylindrical-Conical Implant with a Progressive Thread Design at Five-Year Follow-Up: A Single-Center Cross-Sectional Retrospective Study

**DOI:** 10.3390/dj14090594

**Published:** 2026-09-14

**Authors:** Jörg-Martin Ruppin, Tobias Graf

**Affiliations:** 1 Implantatzentrum Dr. Ruppin & Kollegen, Private Practice, 82377 Penzberg, Germany; dr.ruppin@iz-ruppin.de; 2 Department of Prosthetic Dentistry, LMU University Hospital, LMU Medizin, Ludwig-Maximilians-Universität München, Goethestraße 70, 80336 Munich, Germany

**Keywords:** dental implants, five-year follow-up, peri-implant health, functional implant prosthodontic score FIPS, marginal bone level

## Abstract

Objectives: We aim to assess the 5-year clinical outcomes of a cylindrical-conical implant system with a progressive thread design and a sandblasted, acid-etched surface and its fixed prostheses under routine clinical conditions. Methods: This retrospective single-center cross-sectional follow-up study included patients treated with implants with a progressive thread design and an internal butt-joint connection between January 2019 and August 2020 at a specialist referral practice in Germany. Of 112 patients treated, 44 (94 implants with fixed restorations) attended the five-year recall. Outcomes were the Functional Implant Prosthodontic Score (FIPS), peri-implant tissue health parameters (bleeding on probing, suppuration, dehiscence, width of keratinized mucosa, and other signs of inflammation), marginal bone level on periapical radiographs, and implant survival. Results: All 94 implants available for examination remained in situ at five years, corresponding to 100% survival among recalled implants. Mean FIPS was 7.6 out of 10 (interproximal 1.3, occlusion 1.8, design 1.6, mucosa 1.4, bone 1.5). Bleeding on probing was absent in 37.2% of implants and present at ≥3 of 6 sites in 30.9%; suppuration was absent in all cases. Bleeding was descriptively more frequent where keratinized mucosa was narrow: implants with <2 mm bled at nearly three times as many sites as those with ≥2 mm. No implant showed a marginal bone level ≥2.0 mm apical to the shoulder. Conclusions: Within the limitations of this retrospective cross-sectional follow-up study, the implant system with a progressive thread design demonstrated favorable 5-year prosthodontic, biological and radiological findings under referral-practice conditions.

## 1. Introduction

Tooth retention in adults has improved in recent decades; population oral health surveys report less tooth loss and better self-care, even as the number of dental implants placed each year continues to rise [1,2]. Osseointegrated implants are a well-established option for replacing missing teeth, with longitudinal studies consistently reporting ten-year survival above 95% across different implant systems and loading protocols [3,4,5,6,7,8].

Clinical practice, however, increasingly demands predictable performance under challenging conditions—reduced bone quality, previously augmented sites, and protocols permitting early or immediate restoration—which depend critically on high primary stability and implant macro-design. Survival alone is an inadequate endpoint. Recent assessments emphasize peri-implant tissue health, marginal bone stability, and patient-relevant functional outcomes. Recognizing that implants may remain in situ despite exhibiting biological or prosthodontic deficiencies, extended outcome measures and refined case definitions for peri-implant diseases have accordingly become central to long-term assessment [9,10,11].

Crestal bone behavior is shaped by both implant macro-design and collar configuration. Peri-implant bone responds to the apico-coronal position of the rough–smooth interface and to the presence and height of a machined collar, and preclinical work with nonsubmerged implants of the same family has shown that different machined-collar lengths yield distinct patterns of crestal remodeling—support for the rationale behind short, machined collars [12]. This matters most when implants are placed at or slightly above the crest under minimally invasive protocols, where early bone–implant contact and soft-tissue stability are decisive.

Whether such stability is maintained depends on implant-, tissue-, patient-, and prosthodontics-related factors. The 2017 World Workshop on the Classification of Periodontal and Peri-Implant Diseases defined peri-implantitis as bleeding on probing and/or suppuration together with progressive radiographic marginal bone loss relative to baseline; in the absence of baseline radiographs, a bone level ≥ 3 mm apical to the implant shoulder combined with bleeding on probing is considered as diagnostic proof [11].

These conditions are common—peri-implant mucositis affects roughly one-third to one-half of implants, and peri-implantitis occurs in about 8–14% after a few years, depending on definition and population [9,13]. For a two-piece implant system with an internal tube-in-tube connection, mucositis and peri-implantitis prevalences of 35.6% and 7.6% at the implant level (41.6% and 13.9% at the patient level) have been reported after a median of 23 months. These data underline how common biological complications are in daily practice [14].

Beyond implant- and patient-related factors, prosthodontic design is an important, modifiable determinant of peri-implant health. The emergence angle and subgingival contour of implant-supported restorations have been implicated in plaque accumulation, soft-tissue inflammation, or marginal bone remodeling [15,16,17,18]. Preclinical and histological work links wide emergence angles and overly convex subgingival profiles to greater marginal bone loss and disruption of the junctional epithelium at the implant supracrestal complex [16,17], while clinical and scoping data indicate that unfavorable emergence profiles correlate with greater probing depths, bleeding on probing, and radiographic bone loss [15,18]. The emergence profile is thus a clinically accessible variable that may influence long-term peri-implant stability around otherwise well-integrated implants.

The width of keratinized mucosa (KM) is another parameter relevant to peri-implant health and patient comfort [19]. Systematic reviews suggest that reduced KM width is frequently associated with increased plaque accumulation, mucosal inflammation, soft-tissue recession, and patient-reported discomfort during oral hygiene, particularly plaque control is suboptimal [20,21]. The lack of KM may itself be a risk factor for peri-implantitis and for more severe mucositis [21]. Despite heterogeneous evidence, the independent impact of keratinized mucosa width on the stability of hard and soft tissues remains a subject of debate [20,21]. In a specialist referral setting, where patients often present with complex anatomical situations and prior augmentation, KM width may be especially relevant for maintaining peri-implant health over time.

Prosthodontic quality can be assessed using the Functional Implant Prosthodontic Score (FIPS), which rates interproximal contacts, occlusion, design, mucosa, and bone at the level of the restoration [22]. Developed and validated for single-unit implant crowns only, FIPS offers a pragmatic, structured measure that, combined with standardized peri-implant disease criteria, supports an integrated view of the biological and functional status of implants [22,23].

Implant systems designed to improve insertion behavior and primary stability—progressive thread geometries and cylindrical–conical bodies intended for compromised bone and modern treatment protocols—warrant evaluation beyond conventional survival reporting. Controlled trials establish efficacy, but follow-up under routine conditions remains essential to confirm long-term safety and performance [5], and referral-based specialist practices, where surgical, restorative, and maintenance care are often provided by different clinicians, are a particularly relevant real-world setting. Evidence for progressive thread geometries over five years is generally scarce. E.g., only one-year clinical data has so far been published for CAMLOG Progressive-Line implants (Altatec GmbH, Wimsheim, Germany), introduced in 2019 [24],—making medium-term follow-up of this design timely.

The present study evaluated the five-year clinical performance of this implant in a specialist referral practice in Germany. The primary objective was to assess implant survival, peri-implant tissue health, marginal bone level, and prosthodontic quality (FIPS); secondary objectives were to explore associations of keratinized mucosa width and restoration emergence profile with peri-implant tissue status at recall. To our knowledge, this is the first medium-term (five-year) clinical evaluation of this cylindrical–conical, progressive-thread implant. In addition, it is the first for this implant geometry to report real-world referral-practice data, in which surgical, restorative, and maintenance care are delivered by different clinicians.

## 2. Materials and Methods

### 2.1. Study Design and Setting

This retrospective, single-center, cross-sectional follow-up study was conducted at a specialist referral practice for oral implantology (Implantatzentrum Dr. Ruppin und Kollegen, Bichler Str. 17, 82377 Penzberg, Germany). The study was reviewed by the Ethics Committee of the Bavarian State Medical Association (Bayerische Landesärztekammer (BLÄK), reference number 2025-1078), which determined that formal ethical approval was not required for this retrospective analysis. The study was conducted in compliance with the Declaration of Helsinki. All patients provided informed consent for the use of their clinical data. Following surgical treatment, patients were routinely referred to their restorative dentists for prosthodontic rehabilitation and long-term maintenance care.

### 2.2. Patient and Implant Selection

The study cohort included all patients who received at least one CAMLOG Progressive-Line implant between January 2019 and August 2020. All treatment steps—planning, augmentation, and exposure—were performed by a single surgeon (J.R.) at the study center, defining a baseline population of 112 patients. No additional inclusion or exclusion criteria were applied; patients meeting this criterion were eligible for the five-year recall if they could be contacted. Details on patient flow and attrition are provided in Section 3.1.

### 2.3. Implant System

All implants placed during the index period were CAMLOG Progressive-Line implants (Altatec GmbH, Wimsheim, Germany). It is a cylindrical-conical Grade 4 commercially pure titanium implant with a blasted and acid-etched surface (Promote Plus^®^), a 0.4 mm machined collar, and a roughened surface extending to within 0.4 mm of the implant shoulder. The body combines a crestal anchoring thread at the neck, a parallel-walled central segment with a saw-tooth thread, and a conical apex threaded to the tip—the progressive thread design—and the implant–abutment interface uses a Tube-in-Tube^®^ internal butt-joint connection. Implant diameters were 3.8, 4.3, and 5.0 mm and lengths 9–13 mm.

### 2.4. Surgical and Prosthetic Protocol

Implants were inserted into healed sites without immediate post-extraction placement, in accordance with a conventional delayed-loading protocol. When necessary, augmentation was performed concurrently with implant placement; this most commonly involved internal sinus floor elevation, followed by lateral bone augmentation, external sinus floor elevation, and block grafting with autologous bone each. The implants underwent either transmucosal or submerged healing. Following an average healing duration of 12 weeks, patients were referred back to their prosthodontists for definitive restoration with fixed crowns or bridges. A minority of implants supported removable restorations; the present analysis focuses on those restored with fixed prostheses.

### 2.5. Clinical and Radiographic Examination

All recall examinations were performed by a single experienced clinician (T.G.) at the study center. The following parameters were recorded per implant:

- Implant status (in situ/failed/not evaluable).
- Percussion sensitivity (negative/positive).
- Functional Implant Prosthodontic Score (FIPS): five subscales (interproximal, occlusion, design, mucosa, bone), each scored 0–2 (maximum total 10) [22]. In this cohort the FIPS was applied at the implant level, with each implant treated as an individual restorative unit; for splinted restorations and 3-unit fixed dental prostheses (FDPs), every supporting implant was scored separately, so that a finding such as chipping was attributed only to the affected abutment. As FDPs were included, the interproximal score was limited to 0 or 2 for terminal single-tooth implants and mesial bridge abutments and was not assessable at terminal bridge abutments. The total FIPS was derived by summing the subscale means. As the cohort also included splinted restorations and 3-unit FDPs, this implant-level application represents a pragmatic, not independently validated modification and is treated as exploratory.
- Bleeding on probing (BOP): recorded as the number of bleeding sites per implant (0–6) on gentle probing at a standardized force of 0.25 N, and categorized, for pragmatic reporting, as 0/6 (healthy), 1–2/6 (possible mild mucositis), or ≥3/6 (clear inflammatory sign).
- Suppuration (Pus): binary (present/absent).
- Mucosal dehiscence: binary (present/absent).
- Width of keratinized mucosa (KM): measured at the mid-buccal aspect and categorized as <2 mm or ≥2 mm.
- Additional signs of inflammation: binary (present/absent).
- Plaque Score (PS): at each of six peri-implant sites (three buccal, three oral), visible plaque or deposits were scored as present or absent based on visual assessment (without disclosing agents), giving a count of 0–6 per implant. Plaque load was categorized per implant as 0/6 (plaque-free), 1–2/6 (low), or ≥3/6 (moderate-to-high), mirroring the bleeding-on-probing categories.
- Periapical radiograph: marginal bone level measured mesially and distally on right-angle periapical radiographs as the distance from the implant shoulder to the most coronal bone-to-implant contact, recorded to the nearest 0.1 mm (a value of 0 mm denotes bone at the implant shoulder). A 0.5-mm threshold was used for categorization and emergence-profile comparison. Radiographs unsuitable for exact measurement were recorded as not evaluable.
- Emergence profile: classified retrospectively on periapical radiographs as concave or convex, immediately coronal to the implant, separately at the mesial and distal aspects; radiographs not suitable for an exact measurement were recorded as not evaluable.

### 2.6. Statistical Analysis

Data were summarized descriptively: continuous variables as mean ± SD, categorical variables as counts and percentages, and marginal bone level by category (0–0.5, 0.5–1.5, ≥1.5 mm, and not evaluable). Given the small, imbalanced subgroups clustered within patients, no formal hypothesis testing was performed, and no *p*-values are reported; all subgroup comparisons are descriptive. Descriptive summaries were computed in Microsoft Excel (Microsoft 365, Version 2606, (Build 20131.20112), Redmond, WA, USA).

Within-patient clustering of bleeding on probing was quantified by an intraclass correlation coefficient from a random-intercept (one-way random-effects) model (ICC = 0.54), confirming that implants within the same patient were not independent. Given this clustering and the small, imbalanced subgroups, keratinized-mucosa comparisons are reported as descriptive point estimates only, without confidence intervals or significance tests, and all findings are regarded as exploratory and hypothesis-generating. The ICC was computed in jamovi (version 2.7; The Jamovi Project, Sydney, Australia), which uses the R statistical environment (R Foundation for Statistical Computing, Vienna, Austria).

## 3. Results

### 3.1. Patient Flow and Implant Attrition

**Figure 1:**
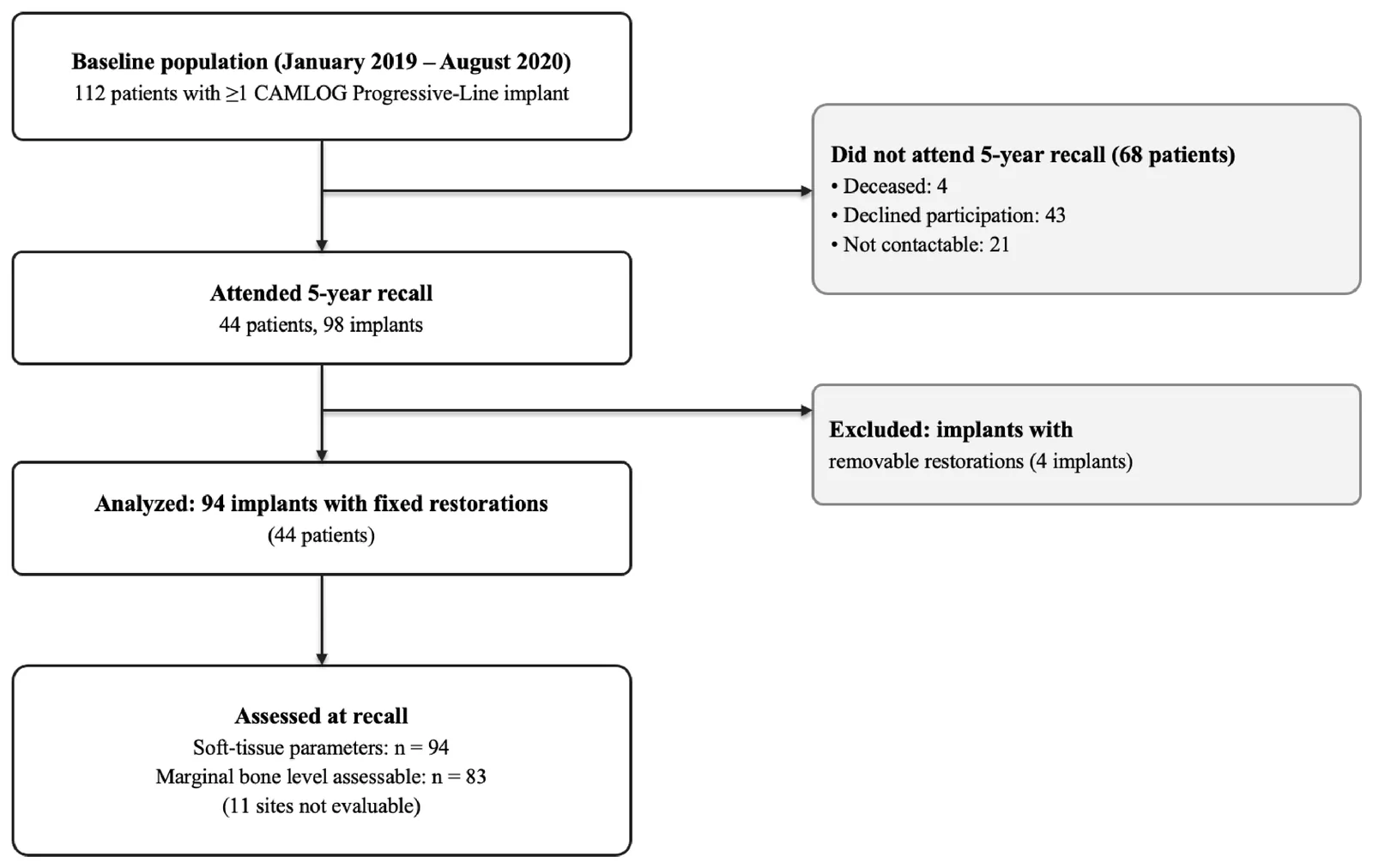
Participant flow (STROBE).

The participant flow is summarized in Figure 1. Among the 112 patients treated from January 2019 to August 2020, 44 individuals, accounting for 94 implants with fixed restorations, participated in the five-year follow-up examination. Of the 68 patients who did not attend, 4 had passed away, 43 declined to participate, and 21 could not be reached; consequently, the implant status of these non-attending patients remains undetermined. All 94 implants available for examination at the five-year recall remained in situ (100%). As implant status was unavailable for non-attenders and no failures were observed among recalls, survival analysis was not performed. Soft-tissue parameters were recorded for all 94 implants, while the marginal bone level was radiographically assessable in 83 cases, with 11 sites not evaluable.

All 94 implants examined were in situ at recall (100%); however, this rate applies only to the 44 patients (39.3% of the baseline population) who attended and cannot be generalized to the full baseline population. Because the implant status of non-attenders is unknown, survival of the complete baseline population cannot be determined. In an extreme worst-case sensitivity analysis assuming that every non-attending patient had experienced total implant failure, patient-level survival would be as low as 39.3%.

### 3.2. Baseline Implant Characteristics

Baseline characteristics of the five-year follow-up cohort (94 implants in 44 patients) are summarized in Table 1. Mean patient age at placement was 58.3 ± 13.1 years; 30 patients were women and 14 were men.

### 3.3. Prosthodontic Outcomes: FIPS

The mean total FIPS was 7.57 out of a maximum of 10. Subscale scores are shown in Table 2, each averaged over the implants assessable for that item. Occlusion scored highest (1.77/2) and the interproximal dimension lowest (1.29/2).

Stratified by restoration type (Table 3), single crowns showed higher subscale means than bridge-supported implants, most notably for the mucosa item (1.70 versus 1.15); the interproximal score was limited by non-assessable terminal bridge abutments (assessable in 25 of 46 bridge implants). These differences are descriptive only, given the small, clustered subgroups.

### 3.4. Peri-Implant Tissue Health

Peri-implant soft tissue parameters are summarized in Table 4. Applying the 2017 World Workshop case definitions, 35 implants (37.2%) were peri-implant healthy (no bleeding on probing) and 59 (62.8%) had peri-implant mucositis (bleeding on probing without bone loss); no implant met the criteria for peri-implantitis, and suppuration was absent throughout. The Plaque Score (PS) values followed bleeding closely: no plaque-free implant bled at three or more sites, whereas 26 of the 36 implants with a high plaque score did, consistent with a plaque-driven inflammatory response.

Subgroup analysis by keratinized mucosa width descriptively indicated increased bleeding in areas of deficient mucosa. Implants with a KM width of less than 2 mm (n = 13) exhibited bleeding at an average of 3.23 ± 1.96 out of six peri-implant sites, compared with 1.35 ± 1.53 sites in implants with ≥2 mm (n = 81), a difference of 1.89 sites. A bleeding-positive status (≥1 site) was observed in 92.3% of implants with less than 2 mm of KM, compared to 58.0% in those with ≥2 mm, representing a difference of 34.3%. Implants with less than 2 mm of KM were found in both the maxilla (9/78 [11.5%]) and the mandible (4/16 [25.0%]). Given the small, imbalanced, and patient-clustered subgroups, these differences are descriptive and exploratory only, and no inferential estimates (confidence intervals) were derived.

### 3.5. Marginal Bone Level

Marginal bone levels are summarized in Table 5.

Of 83 radiographically evaluable implants per site (11 were not evaluable owing to recording technique, angulation or superimposition) the bone level was less than 0.5 mm of the implant shoulder in 56 implants (67.5%) mesially and 46 (55.4%) distally. No implant reached a marginal bone level of 2.0 mm.

As no T0 baseline radiograph with prostheses to compare was available, these figures represent a cross-sectional measurement of the marginal bone level at five years, not bone loss calculated from baseline.

### 3.6. Prosthodontic Emergence Profile

In 92 out of 94 implants, the emergence profile was assessable on periapical radiographs at each proximal aspect: mesially, 74 were concave and 18 convex; distally 76 concave and 16 convex. Because marginal bone level was categorized using a 0.5 mm threshold, profiles were compared by the proportion of implants exceeding this threshold. This proportion was approximately doubled for convex profiles (mesial: 50% vs. 25%; distal: 67% vs. 37%), whereas bleeding on probing did not differ by profile.

Given the marked group imbalance and the clustering of implants within patients, these observations are descriptive only and should be read as a cautious tendency rather than an association.

Table 6 summarizes the associations between emergence profiles and marginal bone levels, as well as bleeding on probing (BOP). Although a higher proportion of convex profiles exceeded the threshold, this was not associated with a difference in BOP.

## 4. Discussion

After five years, the cylindrical–conical implant demonstrated satisfactory biological and prosthodontic outcomes under routine referral conditions. All 94 implants examined remained in situ, and none met the 2017 World Workshop case definition for peri-implantitis [11]. Both FIPS and marginal bone levels were consistent within the range reported for contemporary implant systems, and in line with the study objectives. Given that the status of non-attending patients is unknown, this finding describes the examined cohort rather than the original population.

The absence of any failures is consistent with the >95% ten-year survival documented across implant systems and protocols [3,4,5,6,7]. The absence of peri-implantitis places this cohort at the favorable end of a range in which the disease affects roughly 8–14% of implants within a few years [9,13]. Part of this likely reflects attendance bias, as patients who return for recall tend to be more adherent and hygiene-conscious, so the true disease burden may be underestimated.

Bleeding on probing was common but mostly limited: about one-third of the implants exhibited bleeding at three or more sites, another third at one or two sites, while the remainder showed no bleeding. The ≥3/6 rate is largely consistent, though somewhat lower than the 41.6% prevalence of mucositis reported for the same tube-in-tube connection [14]. This places our cohort within the expected range rather than marking a center-specific anomaly. Bleeding without concomitant bone loss falls within the potentially reversible spectrum of mucositis. The clearest gradient was observed for keratinized mucosa width; implants with less than 2 mm exhibited bleeding at nearly three times as many sites, in line with reports of a protective soft-tissue band against provoked bleeding [19,20].

Among prosthetic factors, the subgingival emergence profile has been linked to peri-implant outcomes: preclinical work associates wide or convex profiles with greater marginal bone loss and disruption of the junctional epithelium at the supracrestal complex [16,17] and clinical and scoping data associate unfavorable profiles with deeper probing depths, more bleeding, and greater radiographic bone loss [15,18,25]. In our data, convex profiles showed roughly double the proportion of implants exceeding the radiographic measurement threshold, whereas bleeding did not differ by profile. This bone-level tendency is directionally consistent with the literature, but the convex subgroup was small and clustered, and no relationship with bleeding was seen; the observation should therefore be read as a cautious, exploratory finding rather than a confirmed association.

Crestal bone sat within 0.5 mm of the shoulder in most implants, and none extended to 2.0 mm, which aligns with the design rationale that a short (0.4 mm) machined collar and crestal anchoring thread promote early bone–implant contact near the crest. This finding is supported by preclinical evidence [12] and is particularly noteworthy given that most implants were placed in soft, augmented bone, yet none failed. The outcome may be consistent with the role of the progressive thread design in ensuring primary stability [24]. The moderate insertion torque and the absence of immediate placements, however, preclude any inference about immediate protocols. These findings illustrate a cross-sectional status at five years, rather than longitudinal bone loss. Prospective studies provide context by reporting a mean five-year marginal bone loss of approximately 0.2–0.5 mm across common implant systems [26], with augmented sites exhibiting higher values, around 1.9 mm [27]. Given that our cohort was predominantly augmented and lacked a baseline, these values are not directly comparable. Nonetheless, our cross-sectional levels fall within the lower range reported for such sites.

Prosthodontically, a mean FIPS of 7.6/10, applied at the implant level, indicates solid integration: occlusion scored highest—expected for fixed restorations in the relatively controlled posterior-maxillary loading environment, while the interproximal subscale was lowest. This likely reflects a proximal drift in long-standing restorations. Comparable prosthodontic performance has been reported for implant-supported restorations over shorter observation periods [28,29,30]. Therefore, the current five-year data extend these data to a longer interval, albeit descriptively.

Several limitations qualify our findings. The study is retrospective and single-center with no control group. The cohort, predominantly posterior maxillary implants placed in augmented bone and referred to a specialist practice, is not representative of the wider implant-treated population, so generalization should be made with caution. Attrition was substantial, and non-attenders may differ systematically from attenders in terms of complications, general health, or oral hygiene behavior. Although the reasons for non-return are documented, residual selection bias cannot be excluded. The classification of emergence profiles using periapical radiographs is limited to capturing the mesiodistal dimension of a three-dimensional contour. This method does not evaluate buccal or lingual convexity, which are areas where plaque accumulation and its effects are most pronounced. Consequently, this approach serves as a practical approximation confined to the proximal dimension. The FIPS was utilized at the implant level, representing a pragmatic adaptation of the original single-crown score, which has not undergone independent validation. Because the FIPS is validated only for single-unit crowns, its implant-level application here should be regarded as exploratory. The plaque score was not recorded by staining or disclosing agents, so it is exploratory and might underestimate plaque. Finally, the absence of baseline radiographs at prostheses delivery means peri-implant bone is reported as a status at five years rather than as a change over time. As restorative and maintenance care was delivered by different referring dentists, the findings also reflect real-world referral-practice conditions and provide descriptive medium-term data on fixed restorations—evidence that remains scarce in the literature and could serve as a baseline for future follow-up of the same cohort.

Clinical Relevance: In a practical referral context, this cylindrical-conical, progressive-thread implant demonstrated favorable prosthodontic and biological outcomes over a five-year period, including in previously augmented sites. A narrow zone of keratinized mucosa (<2 mm) was descriptively accompanied by more bleeding on probing. These observations may support closer clinical monitoring and the need for further prospective confirmation.

Moreover, convex subgingival emergence profiles were descriptively accompanied by a higher proportion of implants exceeding the marginal bone measurement threshold (a cross-sectional observation), without a corresponding increase in bleeding, which may support the importance of controlled restoration contours.

As all these findings are cross-sectional and hypothesis-generating, they should inform clinical monitoring rather than be regarded as confirmatory evidence.

## 5. Conclusions

Within the limitations of this retrospective study, favorable cross-sectional findings were observed at the five-year recall among the examined implants and their superstructures under routine referral conditions. All implants examined at recall remained in situ (100% survival in the recalled cohort); none met the 2017 World Workshop case definition for peri-implantitis. Both prosthodontic quality (FIPS) and marginal bone levels were comparable to values reported for contemporary implant systems. More pronounced peri-implant bleeding was descriptively more frequent with a narrow zone of keratinized mucosa. This underscores the clinical importance of soft-tissue conditions. Prospective studies with standardized baseline records are needed to confirm these findings.

## Figures and Tables

**Table 1:** Baseline characteristics of the five-year follow-up cohort (n = 94 implants, n = 44 patients).

Parameter	Value
**Patient-level (n = 44)**	
**Women/men**	30 (68.2%)/14 (31.8%)
**Age at placement, years (mean ± SD)**	58.3 ± 13.1 (range 19–84)
**Implant-level (n = 94)**	
**Insertion torque, Ncm (mean ± SD)**	27.2 ± 8.0 (range 15–45; n = 76)
**Bone quality, D1/D2/D3/D4**	2 (2.1%)/16 (17.0%)/41 (43.6%)/35 (37.2%)
**Augmentation performed**	82 (87.2%)
** Internal sinus floor elevation**	49 (52.1%)
** Lateral bone augmentation (bone chips)**	16 (17.0%)
** External sinus floor elevation**	13 (13.8%)
** Block augmentation**	3 (3.2%)
** Combined techniques**	9 (9.6%)
** Bone spreading**	1 (1.1%)
**Transmucosal healing**	56 (59.6%)
**Submerged healing**	38 (40.4%)

**Table 2:** Functional Implant Prosthodontic Score (FIPS) at five-year follow-up. Each subscale is the mean of the implants assessable for that item (column n). Denominators differ because the interproximal score is not assessable at terminal bridge abutments and individual items were occasionally not evaluable, for example, the bone item where no assessable radiograph was available.

FIPS Subscale	Maximum Score	Mean Score (5-Year)	n
**Interproximal**	2	1.29	68
**Occlusion**	2	1.77	90
**Design**	2	1.60	94
**Mucosa**	2	1.43	94
**Bone**	2	1.48	87
**Total FIPS**	10	7.57	—

**Table 3:** Functional Implant Prosthodontic Score (FIPS) subscales by restoration type at five-year follow-up (descriptive; each value is the mean of assessable implants, column n).

Restoration Type	Interproximal	Occlusion	Design	Mucosa	Bone	Total FIPS
Single crown (48 implants)	1.30 (43)	1.81 (47)	1.68 (47)	1.70 (47)	1.54 (41)	**8.03**
Fixed dental prosthesis/bridge (46 implants)	1.28 (25)	1.72 (43)	1.52 (47)	1.15 (47)	1.43 (46)	**7.11**
All fixed restorations (94 implants)	1.29 (68)	1.77 (90)	1.60 (94)	1.43 (94)	1.48 (87)	**7.58**

**Table 4:** Peri-implant soft tissue parameters at five-year follow-up (n = 94 implants).

Parameter	n	%
**Bleeding on probing (BOP) (n = 94)**		
**BOP 0/6 (healthy)**	35	37.2%
**BOP 1–2/6 (possible mild mucositis)**	30	31.9%
**BOP ≥ 3/6 (clear inflammatory sign)**	29	30.9%
**Soft-tissue findings**		
**Suppuration (Pus)**	0	0%
**Mucosal dehiscence**	7	7.5%
**Keratinized mucosa < 2 mm**	13	13.8%
**Additional signs of inflammation**	4	4.3%
**All parameters negative**	47	50.0%
**Plaque (n = 94)**		
**Plaque 0/6 (plaque-free)**	25	26.6%
**Plaque 1–2/6 (low)**	33	35.1%
**Plaque ≥3/6 (moderate–high)**	36	38.3%

**Table 5:** Distribution of marginal bone level at five-year follow-up (n = 83 evaluable implants per site; percentages of evaluable).

Marginal Bone Level Category	Mesial n (%)	Distal n (%)
0–0.5 mm	56 (67.5%)	46 (55.4%)
0.5–1.5 mm	25 (30.1%)	32 (38.6%)
≥1.5 mm	2 (2.4%)	5 (6.0%)
Not radiographically evaluable	11	11
Total implants	94	94

**Table 6:** Emergence profile versus marginal bone level and bleeding on probing, by proximal aspect (descriptive).

Aspect	Profile	n	Bone Level > 0.5 mm	BOP ≥ 3/6
Mesial	Concave	74	25% (17/67)	30% (22/74)
Mesial	Convex	18	50% (8/16)	28% (5/18)
Distal	Concave	76	37% (25/68)	30% (23/76)
Distal	Convex	16	67% (10/15)	25% (4/16)

## Data Availability

The data that support the findings of this study are available upon reasonable request from the corresponding author. The data is not publicly available due to privacy or ethical restrictions.
